# SCID newborn screening: seven-year performance and outcomes including T-cell lymphopenia in Catalonia (Spain)

**DOI:** 10.3389/fimmu.2026.1803232

**Published:** 2026-04-16

**Authors:** Ana Argudo-Ramírez, Andrea Martín-Nalda, Javier Laguna, Jacques G. Riviere, José Manuel González de Aledo-Castillo, Rosa M. López-Galera, Roger Colobran, Laura Batlle-Masó, Yania Quintero, Carmen Martínez, Abraham J. Paredes-Fuentes, Janire Perurena-Prieto, Laura Alonso, Blanca Prats-Viedma, Judit García-Villoria, Pere Soler-Palacín

**Affiliations:** 1 Section of Inborn Errors of Metabolism-IBC, Department of Biochemistry and Molecular Genetics, Biomedical Diagnostic Center (CDB), Hospital Clínic of Barcelona, Barcelona, Catalonia, Spain; 2 Pediatric Infectious Diseases and Immunodeficiencies Unit, Hospital Universitari Vall d’Hebron, Barcelona, Catalonia, Spain; 3 Infection and Immunity Research Group, Vall d’Hebron Research Institute (VHIR), Barcelona, Catalonia, Spain; 4 Institut d’Investigacions Biomèdiques August Pi i Sunyer (IDIBAPS), Barcelona, Catalonia, Spain; 5 Department of Pediatrics, Universitat Autònoma de Barcelona, Barcelona, Catalonia, Spain; 6 Center for Biomedical Research Network on Rare Diseases (CIBERER), Instituto de Salud Carlos III (ISCIII), Madrid, Spain; 7 Immunology Division, Hospital Universitari Vall d’Hebron, Barcelona, Catalonia, Spain; 8 Translational Immunology Research Group, Vall d’Hebron Research Institute (VHIR), Barcelona, Catalonia, Spain; 9 Department of Cell Biology, Physiology and Immunology, Universitat Autònoma de Barcelona, Barcelona, Catalonia, Spain; 10 Department of Clinical and Molecular Genetics, Hospital Universitari Vall d’Hebron, Barcelona, Catalonia, Spain; 11 Department of Pediatric Oncology and Hematology, Hospital Universitari Vall d’Hebron, Barcelona, Catalonia, Spain; 12 Childhood Cancer and Blood Disorders Research Group, Vall d’Hebron Research Institute (VHIR), Barcelona, Catalonia, Spain; 13 Maternal and Child Health Service, Public Health Agency of Catalonia (APSCAT), Department of Health, Generalitat de Catalunya, Barcelona, Catalonia, Spain

**Keywords:** congenital athymia, idiopathic T-cell lymphopenia, lymphopenia, newborn screening, SCID, severe combined immunodeficiency, T-cell receptor excision circles, TREC

## Abstract

**Purpose:**

Severe combined immunodeficiency (SCID) can be detected at birth through T-cell receptor excision circles (TREC) analysis in dried blood spots. This study summarizes the results of the consolidated SCID newborn screening (NBS) program in Catalonia (Spain) during the first seven years of program implementation (2017–2023).

**Methods:**

Newborns were screened for SCID using the EnLite Neonatal TREC assay (cut-off: 20 copies/μL), with confirmatory immunological and genetic testing performed in screen-positive cases. Definitive SCID diagnosis was established according to PIDTC-2022 definitions, while final diagnostic classification followed the recommendations provided by Blom et al.

**Results:**

Among 420,263 screened newborns, 105 screened positive (0.02%). SCID was diagnosed in eight infants and congenital athymia in one, corresponding to an overall incidence of 1:46,753 live births. Identified genetic defects included *RAG2* (n=3), *PNP* (n=1), *IL2RG* (n=2), *DCLRE1C* (n=1), and *FOXI3* (n=1) (one case remained genetically undefined). Six patients underwent early hematopoietic stem cell transplantation, achieving favorable outcomes in five patients, with one death due to post-transplant complications. In addition, gene therapy (*IL2RG*, *DCLRE1C*) and thymic transplantation (*FOXI3*) resulted in successful outcomes in the three remaining patients. Overall and event-free survival reached 88%. Median age at diagnosis was 9 days and median age at initiation of definitive therapy was 3 months. In addition to SCID cases, 53 newborns were diagnosed with non-SCID T-cell lymphopenia (1:7,939), including syndromic, idiopathic, and reversible forms. During follow-up, patients with idiopathic T-cell lymphopenia did not develop significant infectious or autoimmune complications, except for one case of autoimmune neutropenia. Thirty-three cases were classified as false positives (0.008%).

**Conclusions:**

The Catalonian SCID newborn screening program demonstrated high clinical effectiveness, enabling early definitive treatment and excellent survival outcomes with a low false-positive burden. In addition, systematic follow-up of non-SCID T-cell lymphopenia identified through SCID screening appears warranted. These findings support the sustainability and clinical value of universal SCID screening programs.

## 1 Introduction

Newborn screening (NBS) for severe combined immunodeficiency (SCID), a severe inborn error of immunity (IEI), initiated in Wisconsin in 2008. This marked the beginning of SCID screening expansion worldwide (1). Its implementation in Wisconsin was crucial for demonstrating its effectiveness, inspiring others to follow suit (2). Between 2008 and 2017, most US states started implementing NBS for SCID (3), as did several countries, including Taiwan, Israel, and New Zealand (4–6). In 2017, Catalonia (Spain) became the first European region to include SCID in its NBS program (7, 8). This has led other European countries (9) and Spanish regions (Galicia and the Canary Islands in 2023 and the Community of Madrid in 2024), to incorporate SCID into their NBS programs. A recent review by Blom et al. presents an overview of T-cell receptor excision circles (TREC)-based NBS for SCID in Europe (10). The expansion of these programs underscores the relevance of early detection, which has been associated with improved clinical outcomes and survival in newborns with this condition.

TREC quantification is the most commonly used method and has proven to be effective in identifying SCID and other T-cell lymphocyte deficiencies (11). Some countries also use other biomarkers to detect IEIs, such as K-deleting recombination excision circles quantification using molecular biology (12) and/or adenosine (Ado) and deoxyadenosine (dAdo) level testing using tandem mass spectrometry (MS/MS) for adenosine deaminase deficiency (ADA)-SCID screening (13). Furthermore, some countries additionally offer next-generation sequencing (NGS) with a targeted SCID gene panel on dried blood spots (DBS) as a second-tier test following TREC assay (14).

Other non-SCID T-cell lymphopenias (TCLs) can be diagnosed following a positive NBS result for SCID. Blom et al. (15) established recommendations for the terminology to be used in diagnosing SCID and non-SCID TCLs. While SCID is subclassified into typical, leaky, and Omenn syndrome, non-SCID TCL can be classified as reversible conditions, syndromes that include variable T-cell impairment, and idiopathic TCL (iTCL). The authors also highlighted that preterm newborns are more likely to show transiently low TREC values. They provided guidance on which cases are false positives or inconclusive, ensuring accurate classification and reporting. Considering when NBS for SCID was implemented in Catalonia, this manuscript aims to report on the seven-year experience (2017–2023) of NBS for SCID in a region with approximately 8 million inhabitants, which has a single qualified NBS laboratory responsible for analyzing all newborn samples and a single specialized SCID clinical reference unit (CRU) for managing patients with a positive screening result.

## 2 Materials and methods

### 2.1 Study design

Population-based retrospective cohort study with data from January 2017–December 2023 for all newborns screened for SCID in Catalonia, is presented in this manuscript. Definitive SCID diagnoses were made following Primary Immune Deficiency Treatment Consortium 2022 definitions (16), while final diagnoses were established in accordance with Blom et al.’s indications (15). Congenital athymia was diagnosed following European Society for Immunodeficiencies guidelines (17).

We collected data on TREC values, immunological analyses, final diagnosis, treatment, and clinical outcome in newborns referred to the SCID-CRU in these seven years.

### 2.2 NBS laboratory workflow

One NBS laboratory in Hospital Clínic of Barcelona performs the TREC-based SCID screening. A total of 420,263 newborns (55,000–60,000 births/year), has been screened since the program began. Screening samples are collected at 24–72 hours of life. DBS samples are transported daily to the laboratory. Samples with collection time before 24 hours of life or before 7 days post-transfusion, poor DNA amplification, poor quality or insufficient blood quantity were excluded.

NBS for SCID consists of quantifying TREC (copies/μL) using the EnLite Neonatal TREC kit (Revvity, Turku, Finland). DBS testing (1.5 mm diameter spot) was previously described by our group (7). Additionally, since November 2019, Ado and dAdo has been quantified in DBS samples (3.2 mm diameter spot) using MS/MS, with the NeoBase2 Non-derivatized MSMS kit (Revvity, Turku, Finland), particularly to enable the detection of late-onset ADA deficiency.

The current diagnostic decision algorithm for SCID using TREC quantification is shown in **Supplementary Figure 1**. TREC values ≤20 copies/μL (0.6^th^ percentile) in 2/3 tests are used to request a second sample, except with ≤10 copies/μL in term and ≤5 copies/μL in preterm newborns, which are used as cut-offs for referring them directly to the SCID-CRU. In the second sample, ≤20 copies/μL is the cut-off for referral.

For Ado and dAdo quantification, cut-offs were 2.19 µmol/L (99.9^th^ percentile) and 0.17 µmol/L (99.9^th^ percentile), respectively. Positive ADA-SCID cases using this approach are also referred to the SCID-CRU.

### 2.3 SCID-CRU workflow

A single SCID-CRU located at Vall d’Hebron Barcelona Hospital Campus performs confirmation analyses and the subsequent clinical follow-up and treatment of positive cases. When a positive case is identified through NBS, it is immediately reported to the SCID-CRU to contact the family and schedule an emergency visit to the SCID-CRU outpatient clinic within 24–72 hours.

A multidisciplinary team—including a pediatric immunologist, an immunology nurse and a psychologist to address newborn and parent care—conducts this first visit. A detailed medical history is collected; a physical examination is performed; and a blood test is performed including complete blood count, immunoglobulins G, A, M and E, extended T lymphocyte subsets (CD4+ naive cells and recent thymic emigrants), and lymphoproliferation assay in response to phytohemagglutinin (PHA) to confirm or rule out SCID. A urine sample is also collected to rule out congenital cytomegalovirus (CMV) infection, along with purines level testing (inosine, 2-deoxyinosine, guanosine, and 2-deoxyguanosine for PNP deficiency, and adenosine and 2-deoxyadenosine for ADA deficiency were semi-quantitatively evaluated by ultra-high-performance liquid chromatography with ultraviolet detection).

Confirmed SCID cases are placed under protective isolation in the hospital or at home if there are no cohabiting siblings. Prophylaxis, including intravenous or subcutaneous immunoglobulin replacement therapy, is initiated (subcutaneous administration is used in our center in selected patients, as part of our clinical practice, allowing administration of small volumes without the need for venous access), as is antimicrobial prophylaxis with antibiotics, antivirals, and antifungals. Breastfeeding is discontinued until maternal CMV infection is ruled out. In parallel, a custom-designed NGS-based panel including 461 genes associated with primary immunodeficiencies and IEI (**Supplementary Table 1**) is performed while initiating human leukocyte antigen typing and a donor search. Gene selection for the panel was based on the International Union of Immunological Societies (IUIS) classification available during the study period (2022 IUIS classification) (18). The median turnaround time for the NGS results was 45 days (range 10–90 days). In urgent cases, such as patients with suspected SCID, the genetic study is prioritized and results are typically available in less than 20 days.

Close clinical monitoring is performed to detect complications while preparing for curative treatment. In most patients, this involves hematopoietic stem cell transplantation (HSCT), but gene therapy or thymus transplantation may also be considered as treatment options when indicated. The administration of live attenuated vaccines is contraindicated in these patients.

Non-SCID lymphopenia patients are monitored regularly through outpatient visits. The first follow-up is scheduled at three months of age, when blood tests and TREC levels are reassessed. If lymphopenia persists, an array comparative genomic hybridization (CGH) analysis is performed to rule out 22q11.2 deletion syndrome. If the condition continues, a subsequent evaluation at six months includes the NGS-based panel to identify genetic lymphopenia causes, including ataxia-telangiectasia. At this stage, the need for prophylaxis and live attenuated vaccine contraindications are also assessed.

### 2.4 Ethical approval

The study was approved by the Government of Catalonia (Departament de Salut, Generalitat de Catalunya) for its NBS program. Specific informed consent was obtained from all individual participants included in the study whose genetic evaluation was needed. The study was conducted in adherence to the principles outlined in the Code of Ethics of The World Medical Association (Declaration of Helsinki).

### 2.5 Statistical analysis

The database, containing clinical and analytical variables, was created in Microsoft Excel using a standardized data collection sheet on which all variables were recorded. Statistical analysis was performed using GraphPad Prism v8.0.1. Intergroup TREC values were compared using the non-parametric Mann-Whitney U test (19). Differences were considered statistically significant at p<0.05.

## 3 Results

A total of 105 newborns, with a yearly referral of 15 (referral rate: 0.02%, range: 11–20), were referred to the SCID-CRU (64 males, 41 females) during the study period.

In total, 104 newborns were referred due to TREC values below the threshold, and one due to elevated Ado and dAdo levels with normal TREC values. Thirty-five newborns (34%) were urgently referred to the SCID-CRU after an extremely low TREC quantification in the first sample (median age: 12 days [interquartile range (IQR): 8–18]), while the remaining 69 (66%) were referred after second sample confirmation (median age: 24 days [IQR: 17–35]). These timelines reflect the operational workflow of the screening program, including sample collection, transport, laboratory processing, and confirmatory testing. In preterm infants, referral after a second sample may be further delayed due to repeat sampling at corrected gestational age. Importantly, all confirmed SCID cases were identified in the first sample and referred urgently (median: 8 days; range: 3–13 days).

Throughout this period, no false negatives for SCID or congenital athymia were reported.

### 3.1 SCID and congenital athymia

**Figure 1:**
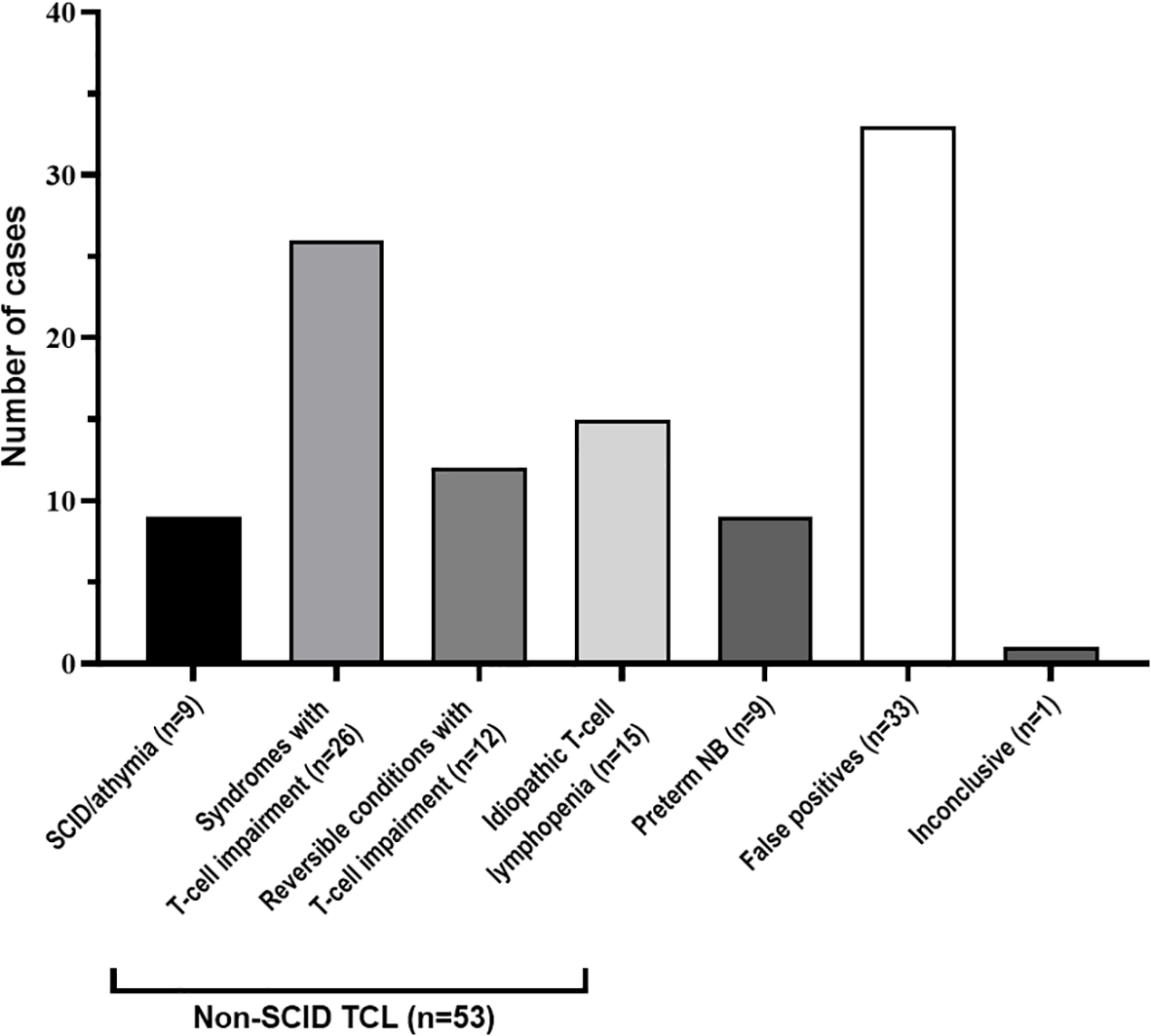
Definitive diagnoses of the 105 newborns referred to the SCID Clinical Reference Unit. NB, Newborns; SCID, Severe combined immunodeficiency; TCL, T-cell lymphopenia.

Eight newborns were diagnosed with SCID and one with congenital athymia (**Figure 1**), giving an overall SCID/congenital athymia incidence of 1 in 46,753 newborns in the seven years of the NBS program. Cumulative incidence over the years is shown in **Table 1**.

**Table 1:** Cumulative incidence of severe combined immunodeficiency (SCID) cases during the study period (2017–2023).

Year	Confirmed diagnoses	Number of newborns analyzed per year	Cumulative number of newborns analyzed	Cumulative incidence
2017	0	66,810	66,810	–
2018	1	64,083	130,893	1:130,893
2019	2	62,061	192,954	1:64,318
2020	0	59,397	252,351	1:84,117
2021	1	57,652	310,003	1:77,501
2022	2	56,466	366,469	1:61,078
2023	3	54,313	420,782	1:46,753

**Figure 2:**
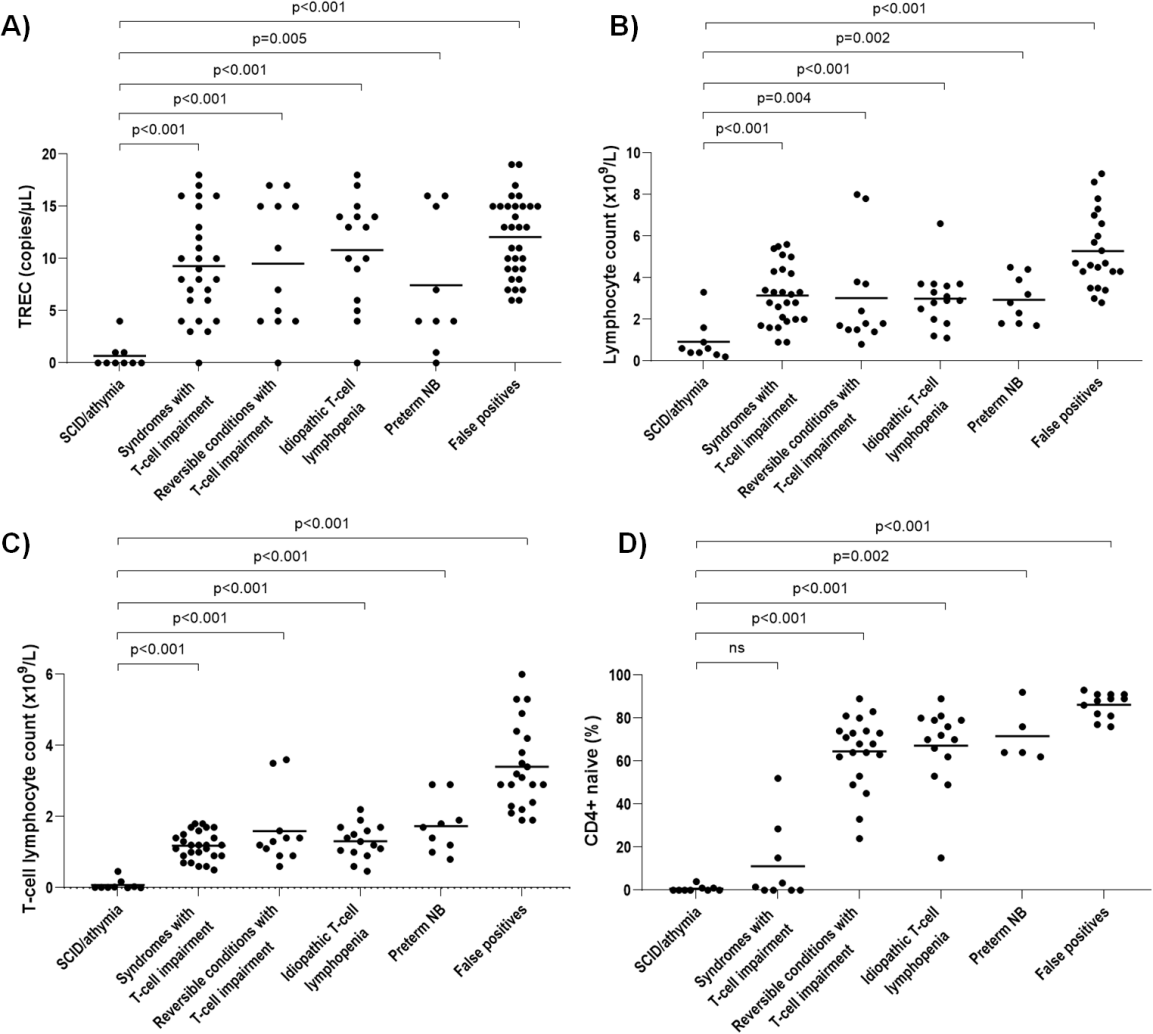
Mean TREC values **(A)**, lymphocyte counts **(B)**, T-cell lymphocyte count **(C)**, and percentage of CD4+ naive cells **(D)**, observed in newborns referred to the SCID Clinical Reference Unit. For the graphical representation of TREC values, for referred newborns with a TREC assay in the first DBS sample, mean values were calculated as the average of the values below the cut-off in that first sample (2 or 3 values). For referred newborns based on the second DBS sample, mean values were calculated as the average of the two values obtained from this second sample. DBS, Dried blood spots; *NB*, Newborns; SCID, Severe combined immunodeficiency; TREC, T-cell receptor excision circles.

Detailed clinical data and TREC values for these nine newborns are shown in **Table 2**. All nine (seven males, two females; gestational age: 35–41 weeks) were referred to the SCID-CRU after TREC analysis on the first DBS sample (no second sample was required) and 8 of 9 newborns had TREC values of 0–1 copies/μL (the other had purine nucleoside phosphorylase [PNP] deficiency and 4 copies/µL). **Figure 2A** shows statistically significant lower TREC values in SCID/athymia newborns compared to those with other diagnoses.

**Table 2:** Demographic data, immunological markers, type of mutation, and treatment of all patients with SCID or congenital athymia.

Case ID	Sex	GA (weeks)	History of consanguinity	TREC (copies/μL) Cut-off ≥20	Lymphocyte count (×10^9^/L) (RI: 3.4–7.6 )	T-cell lymphocyte count (×10^9^/L) (RI: 1.85–5.96)	Lymphocyte phenotype	CD4+ naive (%) (RI: 54–80)	PHA lymphocyte proliferation (RI: >50% control)	Affected gene (transcript)	Zygosity and genetic variants
Case 2018-1	Male	35	No	0	0.4	0.17	T-B-NK+	3.4	Absent (2% of the control)	Not detected	–
Case 2020-1	Female	37	Yes	1	0.4	0.01	T-B-NK+	0	Absent (5% compared to the control)	*RAG2* (NM_000536.4)	HOM: c.1338C>G/p.Cys446Trp
Case 2020-4	Male	40	Yes	4	0.9	0.46	T-B-NK+	52	Decreased (45% compared to the control)	*PNP* (NM_000270.4)	HOM: c.602A>G/p.Glu201Gly
Case 2021-17	Male	38	Yes	0	0.6	0.02	T-B-NK+	15	Absent (10% compared to the control)	*RAG2* (NM_000536.4)	HOM: c.1247G>T/p.Trp416Leu
Case 2022-1	Male	39	No	0	3.3	0.03	T-B+NK-	28.5	Decreased (30% compared to the control)	*IL2RG* (NM_000206.3)	HEMIZ: c.341G>C/p.Gly114Ala
Case 2022-10	Male	40	No	1	1.6	0.01	T-B+NK+	0	Absent (7% compared to the control)	*FOXI3* (NM_001135649.3)	HET: c.230del/p.Gly77Alafs*63
Case 2023-9	Male	38	Yes	0	0.2	0	T-B-NK+	0	Absent	*RAG2* (NM_000536.4)	HOM: c.1247G>T/p.Trp416Leu
Case 2023-13	Male	41	No	0	0.3	0.01	T-B+NK-	1.5	Absent (6% compared to the control)	*IL2RG* (NM_000206.3)	HEMIZ: c.676C>T/p.Arg226Cys
Case 2023-14	Female	37	No	1	0.6	0	T-B-NK+	0	Absent	*DCLRE1C* (NM_001033855.3)	COMP HET: Deletion of exons 1-3 + c.362T>C/p.Met121Thr

COMP HET, compound heterozygous; GA, gestational age; HEMIZ, hemizygous; HET, heterozygous; HOM, homozygous; PHA, phytohemagglutinin; RI, reference interval; TREC, T-cell receptor excision circle.

Total lymphocyte and T-cell counts were consistently low (**Figure 2B, C**). The percentage of naive CD4+ T cells was most often nearly null (median=1.5% [range: 0–52]); the PNP deficiency patient showed 52% naive CD4+ T cells (**Figure 2D**). Consistent with those results, T-cell proliferative responses to phytohemagglutinin (PHA) were absent or markedly reduced.

**Figure 3:**
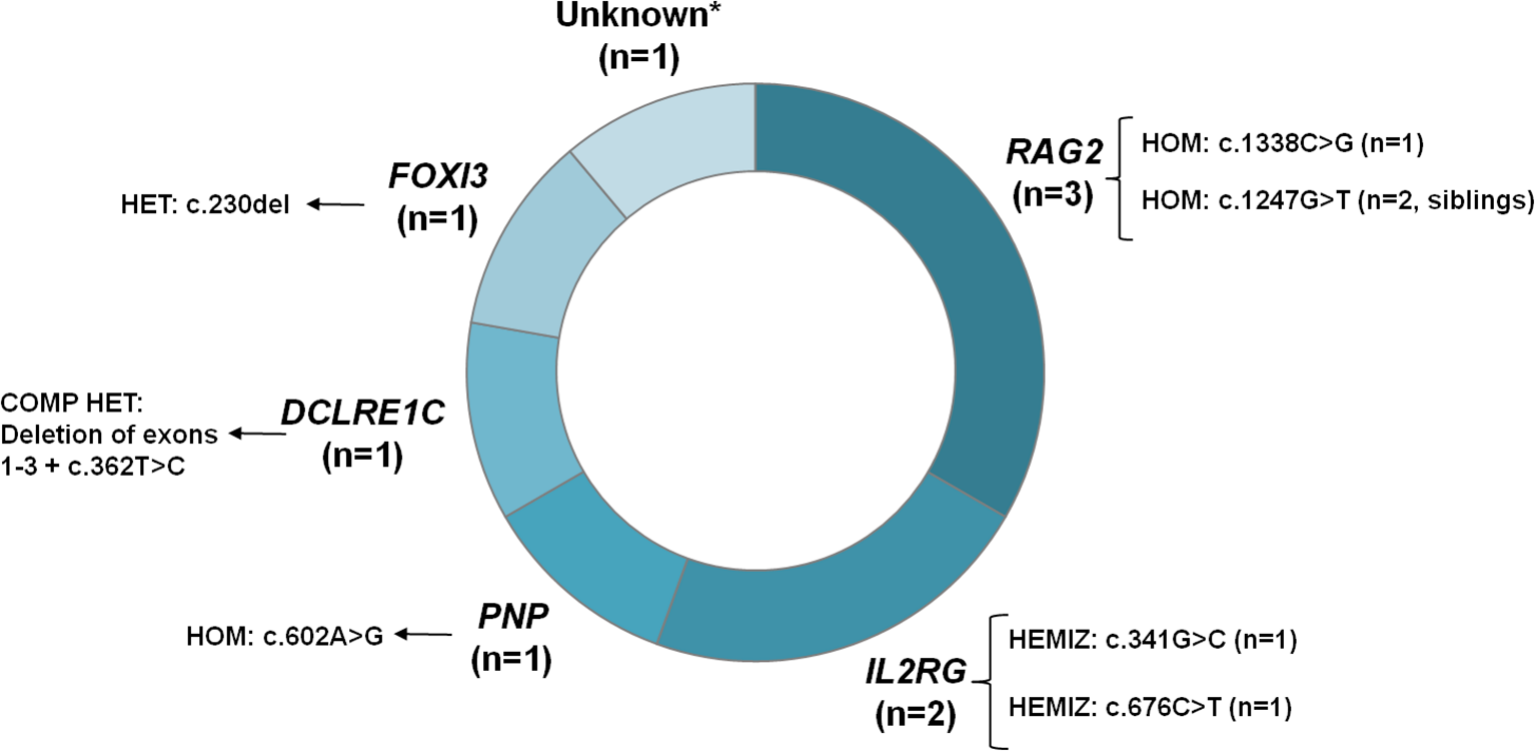
Frequency of altered genes identified in the study. *The studies performed in the patient with no identified genetic cause included array CGH, an NGS-based panel, whole-exome sequencing and whole genome sequencing. CGH, Comparative genomic hybridization; COMP HET, Compound heterozygous; HEMIZ, Hemizygous; HET, Heterozygous; HOM, Homozygous; NGS, Next-generation sequencing.

Genetic disease-causing variants are shown in **Table 2** and **Figure 3**. The most frequently mutated gene was *RAG2* (n=3), with two siblings (cases 2021–17 and 2023-9) carrying the same homozygous variant (c.1247G>T; p.Trp416Leu), and another newborn harboring a different homozygous variant (c.1338C>G, p.Cys446Trp). Two patients had *IL2RG* variants in hemizygous state; and *PNP*, *DCLRE1C* and *FOXI3* mutations were each identified in one case. Additionally, one newborn diagnosed with SCID had an unknown genetic cause. There was a reported history of consanguinity in four cases: the three with *RAG2* gene variants and the patient with a *PNP* gene variant.

Median age at diagnosis was 9 days and median age at initiation of definitive therapy was 3 months (**Table 3**): HSCT in six cases (all before 3 months of age; median age: 75 days); gene therapy in two patients (one *DCLRE1C* mutation, one *IL2RG* mutation); and thymus transplantation in the *FOXI3* mutation patient. Among the six children who underwent HSCT, two received grafts from HLA-matched sibling donors, three from unrelated cord blood donors, and one from a haploidentical family donor. All patients underwent transplantation with conditioning chemotherapy; none received unconditioned transplants. Conditioning regimens were selected according to the IEWP–EBMT guidelines (20). Five patients received a busulfan-based regimen with target exposure of approximately 60 mg·h/L (protocol C), whereas one patient with suspected DNA-repair disorder received cyclophosphamide and fludarabine. All patients also received serotherapy.

**Table 3:** Curative treatment and outcome of all patients affected by SCID or congenital athymia.

Case ID (affected gene)	Treatment	Infections before the treatment	Age at treatment (months)	Donor (in the event of HSCT)	Conditioning	Outcome and current status
Case 2018-1 (unknown)	HSCT	No	3	UCB	ATG 10 mg/Kg, Cyclophosphamide 40 mg/kg, Fludarabine 160 mg/m^2^	Alive 7.5 years after HSCT, off immunosuppression and IgRT
Case 2020-1 (*RAG2*)	HSCT	No	2	MSD	ATG 7.5 mg/kg, Fludarabine 180 mg/m^2^, Busulfan 60 mg·h/L	Alive 6 years after HSCT, off immunosuppression, requires IgRT
Case 2020-4 (*PNP*)	HSCT	No	2.5	Haploidentical	ATG 10 mg/kg, Fludarabine 180 mg/m^2^, Busulfan 58 mg·h/L	Alive 5 years after HSCT, off immunosuppression and IgRT
Case 2021-17 (*RAG2*)	HSCT	No	2.5	MSD	ATG 7.5 mg/kg, Fludarabine 180 mg/m^2^, Busulfan 59 mg·h/L	Death at 9 months post-HSCT: *Klebsiella pneumoniae* Sepsis and refractory hemolytic anemia
Case 2022-1 (*IL2RG*)	GT	No	5	NA (GT)	Low-dose busulfan	Alive 3.4 years after GT, off immunosuppression and IgRT
Case 2022-10 (*FOXI3*)	TT	No	6	NA (TT)	ATG 2 mg/kg, methylprednisolone and cyclosporine	Alive 3 years after TT, off immunosuppression and IgRT
Case 2023-9 (*RAG2*)	HSCT	No	2	UCB	ATG 7.5 mg/kg, Fludarabine 160 mg/m^2^, Busulfan 59 mg·h/L	Alive 2.2 years after HSCT Engrafted with no active complications GvHD prophylaxis with cyclosporine and off IgRT
Case 2023-13 (*IL2RG*)	HSCT	No	3	UCB	Lymphoglobuline 21 mg/kg, Fludarabine 160 mg/m^2^, Busulfan 61 mg·h/L	Alive 2 years after HSCT Engrafted with no active complications GvHD prophylaxis with cyclosporine and off IgRT
Case 2023-14 (*DCLRE1C*)	GT	No	5	NA (GT)	Low-dose busulfan	Alive 14 months after GT, off immunosuppression and IgRT

*ATG*, antithymocyte globulin; *GT*, gene therapy; *GvHD*, graft versus host disease; *HSCT*, hematopoietic stem cell transplantation; *IgRT*, immunoglobulin replacement therapy; *MSD*, matched sibling donor; *NA*, not applicable;  *TT*, thymus transplantation; *UCB*, umbilical cord blood.

No patient experienced infection prior to curative treatment. All showed satisfactory progress during follow-up (range: 9 months–6.5 years), except for one patient who died 9 months after HSCT due to complications from severe post-HSCT hemolytic anemia (overall survival 88%). Currently, no transplanted patient shows signs of graft-versus-host disease or autoimmune complications. All 9 patients experienced normal growth and neurological development (event-free survival 88%), with normalization of TREC and naive CD4+ T cells and replacement immunoglobulin therapy discontinuation in 8/9 patients. This indicates successful immune reconstitution following curative treatment.

### 3.2 Non-SCID TCL

As shown in **Figure 1**, 53 newborns (29 females, 24 males, gestational age: 30–41 weeks) were diagnosed with non-SCID TCL (mean TREC values: 10 copies/μL; range: 0–20 copies/μL; **Figure 2A**), with a non-SCID TCL incidence of 1 in 7,939 newborns for the study period. Of these, 15 newborns (28%) were referred after TREC analysis on the first DBS sample. Based on clinical diagnoses, the cases were classified into three major diagnostic groups following the criteria described by Blom et al. (15): 1) syndromes with T-cell impairment (n=26), 2) reversible conditions with T-cell impairment (n=12) and 3) iTCL (n=15).

In group 1), the most common diagnosis was 22q11.2 deletion/duplication syndrome (13 cases). One of these patients was also diagnosed with ataxia telangiectasia (22q11.2 microdeletion and ataxia telangiectasia simultaneously), and three were independently diagnosed with ataxia telangiectasia. Other conditions in this group included Down’s syndrome (4 cases), *PTEN* gene mutation, SHORT syndrome, and four unclassified syndromic presentations (**Supplementary Table 2**).

Group 2) included eight newborns with congenital chylothorax, three with transient lymphopenia, and one with lymphangiomatosis due to a somatic activating mutation in the *PIK3CA* gene.

In 4/15 patients in group 3), lymphopenia resolved in the first few years of life, with a maximum age of 6 years in the others (**Supplementary Table 3**). Only one patient is being evaluated for autoimmune neutropenia. All patients have grown well, and none have developed severe or recurrent infections during follow-up (median follow-up: 4.3 years [range: 1.5–8 years]). To note, two of these patients carry heterozygous variants in the *AIRE* gene, which were not considered strictly responsible for lymphopenia (21).

### 3.3 Preterm newborns

Nine patients (seven males, two females) had low TREC values due to prematurity. Mean TREC value was 8 copies/μL (range: 0–18 copies/μL; **Figure 2A**). Gestational age ranged from 23–34 weeks, and birth weights from 520–2,065 g. TREC values and immunological studies normalized in these newborns during follow-up (median follow-up: 5.3 years [range: 3–7 years]).

### 3.4 False positives (normal t-cell count)

Thirty-three patients (31%; 22 males, 11 females) were false positives, with an average gestational age of 39 weeks (range: 37–41 weeks) and a mean birth weight of 3,315 g (range: 2,190–4,100 g). Of these, 32 were referred to the SCID-CRU due to low TREC values, with an average of 13 copies/μL (range: 6–20 copies/μL; **Figure 2A**). Although the NBS laboratory initially detected a low TREC value, the SCID-CRU found a normal lymphocyte count, with TREC value normalization at 3–6 months of life.

The other patient was a 2-day-old newborn referred due to elevated Ado and dAdo levels in the first (Ado=12.5 µmol/L; dAdo=0.21 µmol/L) and second (Ado=5.1 µmol/L; dAdo=0.16 µmol/L) DBS samples. The TREC value in the first DBS was normal (57 copies/μL). Subsequent immunological evaluation showed normal lymphocyte subsets. Genetic testing using the NGS-based panel identified two heterozygous variants in the *ADA* gene: one loss-of-function variant (c.320T>C; p.Leu107Pro) and one hypomorphic variant retaining residual enzymatic activity (c.313C>T; p.His105Tyr). This combination was interpreted as benign partial ADA deficiency, which is not strictly considered an IEI.

The false positive rate was 0.008% of all newborns born in the year.

### 3.5 Inconclusive cases

One newborn was classified as inconclusive as they were lost to follow-up, preventing a definitive diagnosis. The infant, born at 38 weeks of gestation, was referred after a second DBS due to abnormal TREC values. However, confirmatory testing and clinical follow-up could not be completed due to loss of contact with the family.

## 4 Discussion

This study evaluates the SCID NBS program in Catalonia seven years after implementation. In this region, there are 55,000–60,000 births/year, with an approximate screening rate of 100% (coverage is considered close to complete, although an exact rate cannot be calculated due to the lack of a centralized registry of live births). Our study results align with findings from other countries with SCID NBS (22–24), demonstrating a comparable SCID incidence (1 in 46,753 live births).

The Catalan SCID NBS program operates within a highly integrated and centralized healthcare structure, which has proven crucial for its effectiveness. One laboratory processes all newborn samples, ensuring standardized analyses. Positive cases are referred to the same SCID-CRU, which provides rapid diagnostic confirmation and treatment initiation. This streamlined model enables prompt multidisciplinary evaluation within 24–72 hours of detection, including advanced immunological testing, genetic analysis, protective clinical measures, and psychological support. Close coordination between the NBS laboratory and SCID-CRU minimizes diagnostic delays and facilitates implementing curative therapies in confirmed SCID/congenital athymia cases. This centralized, collaborative model serves as a best practices example for high-complexity NBS programs.

One of the main aspects under consideration to optimize the SCID NBS program is to adjust the TREC cut-off value. A threshold of ≤20 copies/μL was used for requesting a second sample, while ≤10 copies/μL in term newborns and ≤5 copies/μL in preterm newborns were used for immediate SCID-CRU referral. Although lowering the cut-off could reduce the false-positive rate, true SCID cases could be missed. For instance, data from the Israeli SCID NBS program (same methodology as ours) has raised concerns about decreasing the cut-off (5). Specifically, two confirmed SCID cases in their cohort had TREC values of 18 and 11 copies/μL, which would not have triggered referral under a lower threshold. This emphasizes the need to balance sensitivity and specificity when defining cut-offs, assuming that there are false negatives that cannot be detected through TREC quantification in DBS, such as immunodeficiencies produced by ZAP-70 and MHC-II deficiencies (25, 26), as well as hypomorphic variants in classical SCID-associated genes (such as *RAG1, RAG2*, *IL2RG*, or *ADA*) (27). In our experience, the PNP deficiency patient had the highest TREC values, with 4 copies/µL. In PNP deficiency, progressive T-cell immunodeficiency results from the toxic accumulation of purine metabolites (28). Consequently, immunological parameters such as TREC levels may appear normal in early life (29).

In our cohort, pathogenic *RAG2* gene variants were the most common genetic cause of SCID, affecting three patients (two were siblings). This differs from previous studies in which *IL2RG* pathogenic variants were more common (23, 30), although recent cohort studies have reported an increasing proportion of cases caused by pathogenic variants in *RAG1/RAG2* (31–33). All *RAG2* patients were from consanguineous families. While consanguinity rates in Spain are generally low (3–4%) (34), the overall consanguinity rate in our cohort was 11.4%, rising to 44.4% among patients with SCID/congenital athymia. The ethnic diversity in our cohort may reflect the demographic characteristics of the Catalan population (35), including individuals from regions with higher consanguinity rates. Given the limited number of SCID cases, these findings should be interpreted with caution. Nevertheless, similar patterns have been reported in other cohorts, where *RAG1/RAG2* variants account for a relevant proportion of SCID cases (31–33, 36).

In our study, the most common cause of non-SCID TCL was 22q11.2 deletion/duplication syndrome, found in 13/105 newborns with a positive screening (12%) which concurs with observations in other countries with SCID screening programs (4, 14, 37). This reinforces the importance of performing array CGH early in patients with non-SCID TCL, as established in our CRU follow-up protocol, even in the absence of suggestive phenotypic manifestations.

Regarding other non-SCID TCL cases, those related to reversible causes and syndromic patients are described. Patients with ataxia-telangiectasia (4/105 newborns with positive screening in our study) are considered incidental diagnoses and a challenge for multidisciplinary teams, particularly in terms of how to inform families because it is a serious condition with a substantial emotional impact. Nevertheless, early diagnosis enables promptly identifying potential complications, initiating follow-up care, and providing genetic counselling and psychological support (17, 38).

The NBS program has been critical in identifying patients who require urgent curative treatment such as HSCT, gene therapy, or thymus transplantation. Early protective measures were promptly initiated for all these newborns to prevent infection prior to definitive treatment. These measures include protective in-hospital or at-home isolation; family support; breastfeeding cessation if maternal serology is positive for CMV (IgG+); and prophylaxis with acyclovir, cotrimoxazole, fluconazole, and immunoglobulin replacement therapy. None of the SCID or athymic patients in our study experienced pre-treatment infections, although infections have been reported despite screening (39, 40).

A noteworthy case involved a non-syndromic newborn diagnosed with athymia. Due to low TREC count (1 copy/μL) and a SCID T-B+NK+ phenotype, early intervention, including genetic testing (NGS genetic panel) and protective isolation, was initiated, and an HSCT donor was sought. By day 30 of life, genetic panel results were inconclusive, prompting whole exome sequencing (WES) and array CGH analysis. By day 35 of life, the patient presented with hypocalcemia-induced seizures, raising suspicion of a midline defect and thymic stromal cell defect that was confirmed by *ex vivo* T-cell differentiation studies. WES revealed a frameshift variant in heterozygosity in the *FOXI3* gene (not included in the initial genetic panel), associated with thymic stromal cell defect with a dominant inheritance pattern (41). The patient underwent thymus transplantation at 4 months of age, leading to full immune reconstitution and an excellent clinical outcome. This highlights the value of advanced genetic testing, such as WES, in diagnosing complex cases and the importance of considering congenital athymia in non-syndromic SCID-like presentations.

While HSCT is the primary curative option for most SCID cases (42), two newborns in our study received gene therapy. One patient with X-linked SCID (*IL2RG* gene) and another with SCID due to pathogenic *DCLRE1C* gene variants underwent gene therapy in London and in Paris, respectively. This underscores the need for continued research, novel therapeutic approaches, and international collaboration to ensure wider access to these treatments. With the global implementation of NBS for SCID, we expect an increase in case detection, further emphasizing the importance of advancements in this field.

The incidental diagnosis of iTCL was a challenge in our study. We detected 15 cases (9%) (**Supplementary Table 3**) who did well and did not develop infections or autoimmune complications (only one case with autoimmune neutropenia). However, follow-up was short, and resolution of lymphopenia could not be predicted, raising important questions regarding genetic and functional studies. The lack of established follow-up guidelines for these cases adds uncertainty for families. To date, although studies have been initiated to better define these patients and develop more targeted management strategies, an individualized approach by expert physicians is recommended.

While our study is limited by data from a small region with a low number of screened patients, it benefits from use of a centralized screening laboratory and a specialized clinical reference center. This enables streamlined data collection, uniform clinical practices, and a concentrated pool of expertise, all of which optimize patient evaluation and management.

After seven years of implementation, the SCID newborn screening program in Catalonia demonstrates consistent performance and clear clinical benefit, confirming its capacity to detect both SCID and TCLs in the general population. iTCL lymphopenia remains a clinical challenge in terms of long-term management and prognosis. Our experience provides valuable insights that can help support the expansion of SCID screening programs to other countries and other Spanish regions. Extending this life-saving measure to all newborns would ensure equitable health benefits in all regions. At the time of writing, SCID NBS is undergoing approval in Spain.

## Data Availability

The datasets presented in this study can be found in online repositories. The names of the repository/repositories and accession number(s) can be found in the article/supplementary material.
