# Supplementary material for: SCID newborn screening: seven-year performance and outcomes including T-cell lymphopenia in Catalonia (Spain)
Source: Front Immunol. 2026 Apr 16;17:1803232. doi: 10.3389/fimmu.2026.1803232 (PMC13128370; doi:10.3389/fimmu.2026.1803232)
Supplement: Supplementary file 1 [file DataSheet1.pdf]

Figure S1. TREC Measurements in DBS.

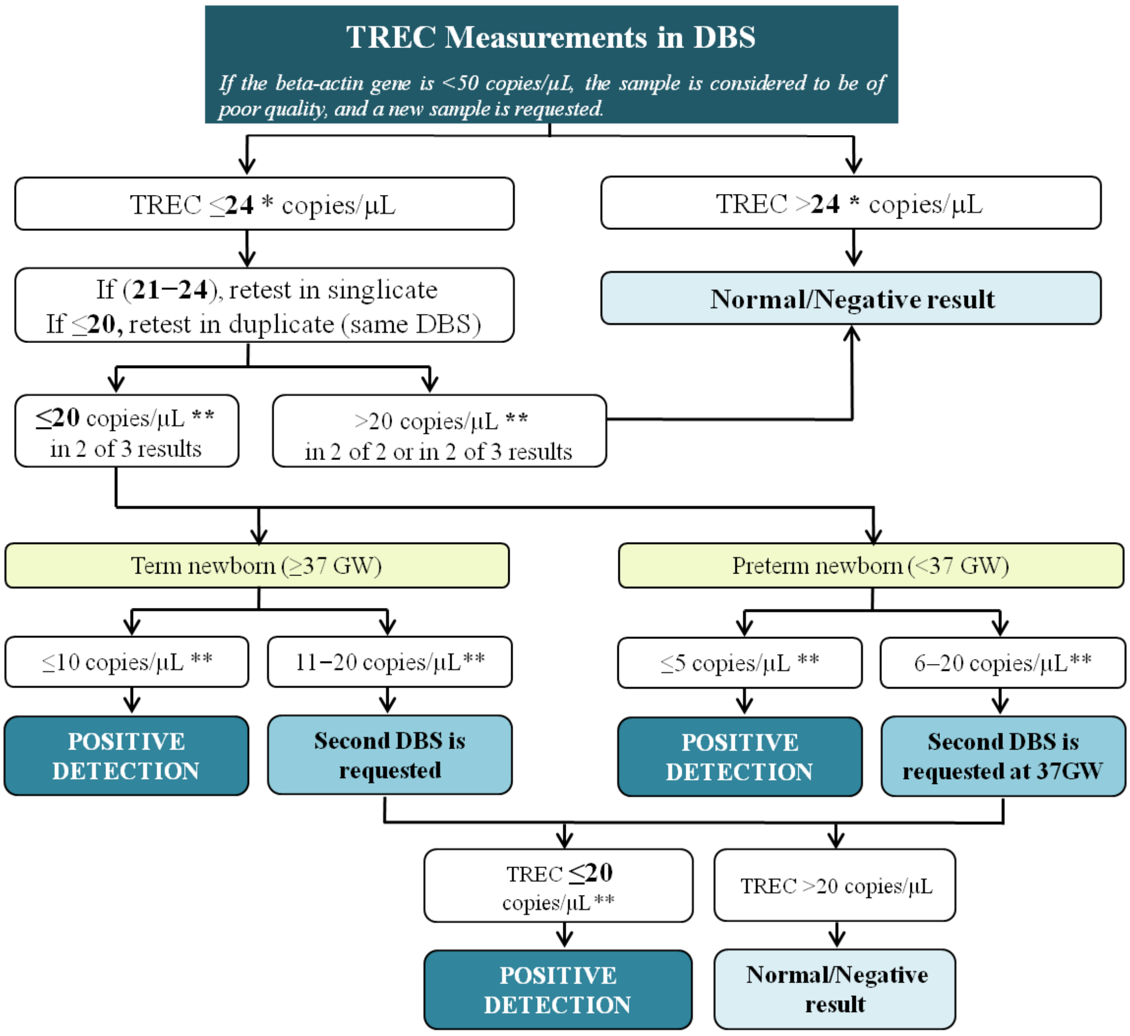

DBS, Dried blood spots; *GW*, Gestational week; *TREC*, T-cell receptor excision circles.

Table S1. List of the 461 genes included in the inborn errors of immunity panel.

ACD	*ACP5*	*ACTB*	*ADA*	*ADA2*	*ADAM17*	*ADAMTS13*	*ADAR*	*AICDA*	*AIRE*	*AK2*
ALPI	*ANGPT1*	*AP1S3*	*AP3B1*	*AP3D1*	*APOL1*	*ARHGEF1*	*ARPC1B*	*ATAD3A*	*ATG4A*	*ATM*
ATP6AP1	*B2M*	*BACH2*	*BCL10*	*BCL11B*	*BLM*	*BLNK*	*BLOC1S6*	*BTK*	*C1QA*	*C1QB*
C1QC	*C1R*	*C1S*	*C2*	*C2orf69*	*C3*	*C4A*	*C4B*	*C5*	*C6*	*C7*
C8A	*C8B*	*C8G*	*C9*	*CARD11*	*CARD14*	*CARD9*	*CARMIL2*	*CASP10*	*CASP8*	*CCBE1*
CD19	*CD247*	*CD27*	*CD28*	*CD3D*	*CD3E*	*CD3G*	*CD40*	*CD40LG*	*CD46*	*CD48*
CD55	*CD59*	*CD70*	*CD79A*	*CD79B*	*CD81*	*CD8A*	*CDC42*	*CDCA7*	*CEBPE*	*CFB*
CFD	*CFH*	*CFHR1*	*CFHR2*	*CFHR3*	*CFHR4*	*CFHR5*	*CFI*	*CFP*	*CFTR*	*CHD7*
CHUK	*CIB1*	*CIITA*	*CLCN7*	*CLEC7A*	*CLPB*	*COPA*	*COPG1*	*CORO1A*	*CR2*	*CRACR2A*
CSF2RA	*CSF2RB*	*CSF3R*	*CTLA4*	*CTNNBL1*	*CTPS1*	*CTSC*	*CXCR2*	*CXCR4*	*CYBA*	*CYBB*
CYBC1	*DBF4*	*DBR1*	*DCLRE1B*	*DCLRE1C*	*DEF6*	*DGAT1*	*DGKE*	*DIAPH1*	*DKC1*	*DNAJC21*
DNASE1L3	*DNASE2*	*DNMT3B*	*DOCK2*	*DOCK8*	*EFL1*	*ELANE*	*ELF4*	*EPG5*	*ERBIN*	*ERCC6L2*
EXTL3	*F12*	*FAAP24*	*FADD*	*FAS*	*FASLG*	*FAT4*	*FCGR1A*	*FCGR2A*	*FCGR2B*	*FCGR3A*
FCGR3B	*FCGRT*	*FCHO1*	*FCN3*	*FERMT1*	*FERMT3*	*FNIP1*	*FOXN1*	*FOXP3*	*FPR1*	*G6PC3*
G6PD	*GATA2*	*GFI1*	*GINS1*	*HAVCR2*	*HAX1*	*HCK*	*HELLS*	*HMOX1*	*HS3ST6*	*HTR1A*
HYOU1	*ICOS*	*ICOSLG*	*IFIH1*	*IFNAR1*	*IFNAR2*	*IFNG*	*IFNGR1*	*IFNGR2*	*IGHM*	*IGKC*
IGLL1	*IKBKB*	*IKBKG*	*IKZF1*	*IKZF2*	*IKZF3*	*IL10*	*IL10RA*	*IL10RB*	*IL12B*	*IL12RB1*
IL12RB2	*IL17F*	*IL17RA*	*IL17RC*	*IL18BP*	*IL1RN*	*IL21*	*IL21R*	*IL23R*	*IL2RA*	*IL2RB*
IL2RG	*IL36RN*	*IL6R*	*IL6ST*	*IL7R*	*INO80*	*IRAK1*	*IRAK4*	*IRF2BP2*	*IRF3*	*IRF4*
IRF7	*IRF8*	*IRF9*	*ISG15*	*ITCH*	*ITGB2*	*ITK*	*ITPKB*	*ITPR3*	*JAGN1*	*JAK1*
JAK2	*JAK3*	*KDM6A*	*KMT2A*	*KMT2D*	*KNG1*	*KRAS*	*LACC1*	*LAMTOR2*	*LAT*	*LCK*
LCP2	*LIG1*	*LIG4*	*LPIN2*	*LRBA*	*LRRC8A*	*LSM11*	*LYST*	*MAGT1*	*MALT1*	*MAN2B2*
MAP1LC3B2	*MAP3K14*	*MAPK8*	*MASP2*	*MBL2*	*MCM10*	*MCM4*	*MEFV*	*MOGS*	*MPO*	*MRTFA*
MS4A1	*MSH6*	*MSN*	*MTHFD1*	*MVK*	*MYD88*	*MYOF*	*MYSM1*	*NBAS*	*NBN*	*NCF1*
NCF2	*NCF4*	*NCKAP1L*	*NCSTN*	*NEIL3*	*NFAT5*	*NFE2L2*	*NFKB1*	*NFKB2*	*NFKBIA*	*NFKBID*

NHEJ1	*NHP2*	*NLRC4*	*NLRP1*	*NLRP12*	*NLRP3*	*NOD2*	*NOP10*	*NOS2*	*NRAS*	*NSMCE3*
OAS1	*ORAI1*	*OSTM1*	*OTULIN*	*PARN*	*PAX1*	*PAX5*	*PDCD1*	*PEPD*	*PGM3*	*PIK3CD*
PIK3CG	*PIK3R1*	*PLCG2*	*PLEKHM1*	*PLG*	*PMS2*	*PNP*	*POLA1*	*POLD1*	*POLD2*	*POLE*
POLE2	*POLR3A*	*POLR3C*	*POLR3F*	*POMP*	*POU2AF1*	*PRF1*	*PRKCD*	*PRKDC*	*PSEN1*	*PSENEN*
PSMA3	*PSMB10*	*PSMB4*	*PSMB8*	*PSMB9*	*PSMG2*	*PSTPIP1*	*PTEN*	*PTPRC*	*RAB27A*	*RAC2*
RAG1	*RAG2*	*RANBP2*	*RASGRP1*	*RBCK1*	*RC3H1*	*RECQL4*	*REL*	*RELA*	*RELB*	*RFX5*
RFXANK	*RFXAP*	*RHOG*	*RHOH*	*RIPK1*	*RMRP*	*RNASEH2A*	*RNASEH2B*	*RNASEH2C*	*RNF168*	*RNF31*
RNU4ATAC	*RNU7-1*	*RORC*	*RPSA*	*RTEL1*	*SAMD9*	*SAMD9L*	*SAMHD1*	*SASH3*	*SBDS*	*SEC61A1*
SEMA3E	*SERPING1*	*SH2D1A*	*SH3BP2*	*SH3KBP1*	*SKIC2*	*SKIC3*	*SLC29A3*	*SLC35C1*	*SLC37A4*	*SLC39A7*
SLC46A1	*SLC7A7*	*SMARCAL1*	*SMARCD2*	*SNORA31*	*SNX10*	*SOCS1*	*SP110*	*SPI1*	*SPINK5*	*SPPL2A*
SRP54	*STAT1*	*STAT2*	*STAT3*	*STAT5B*	*STIM1*	*STING1*	*STK4*	*STN1*	*STX11*	*STXBP2*
SYK	*TAFAZZIN*	*TAP1*	*TAP2*	*TAPBP*	*TBK1*	*TBX1*	*TBX21*	*TCF3*	*TCIRG1*	*TCN2*
TERC	*TERT*	*TET2*	*TFRC*	*TGFB1*	*TGFBR1*	*TGFBR2*	*THBD*	*TICAM1*	*TINF2*	*TIRAP*
TLR3	*TLR7*	*TLR8*	*TMC6*	*TMC8*	*TNFAIP3*	*TNFRSF11A*	*TNFRSF13B*	*TNFRSF13C*	*TNFRSF1A*	*TNFRSF4*
TNFRSF9	*TNFSF11*	*TNFSF12*	*TNFSF13*	*TOM1*	*TOP2B*	*TPP2*	*TRAC*	*TRAF3*	*TRAF3IP2*	*TREX1*
TRIM22	*TRNT1*	*TTC7A*	*TYK2*	*UNC13D*	*UNC93B1*	*UNG*	*USB1*	*USP18*	*VPS13B*	*VPS45*
WAS	*WASF2*	*WDR1*	*WIPF1*	*WRAP53*	*XIAP*	*ZAP70*	*ZBTB24*	*ZNF341*	*ZNFX1*	

Three versions of the panel were used: an initial version including 323 genes (June 2017–June 2021), a second version with 425 genes (July 2021–January 2022), and an updated version implemented in February 2022 comprising 461 genes.

Table S2. Data from first SCID-CRU visit and clinical/immunological outcome of all patients with syndromic lymphopenia.

**Case ID and syndrome**	**Sex**	**GA (weeks)**	**History of consanguinity**	**Infections or other clinical manifestations at the beginning of the study**	**TREC (copies/µL)** Cut-off ≥20	**Lymphocyte count ($\times 10^9$/L)** (RI: 3.4–7.6)	**T-cell lymphocyte count ($\times 10^9$/L)** (RI:1.85–5.96)	**CD4+ T-cell lymphocyte count ($\times 10^9$/L)** (RI:1.14–3.80)	**CD4+ naive (%)** (RI:54– 80)	**PHA lymphocyte proliferation** (RI: >50% control)	**Genetic studies**	**Infections during follow-up**	**Outcome and current status**
Case 2017-5<br><br>22q11.2 deletion	Female	40	No	No	14	4.2	1.7	1.4	NA	Normal (95% of the control)	Array CGH	No	Alive (8 y) without significant complications
Case 2017-8<br><br>22q11.2 deletion	Female	39	No	No	10	5.4	1.6	1.3	NA	Normal (100% of the control)	Array CGH	No	Alive (8 y) without significant complications.
Case 2017-10<br><br>22q11.2 deletion	Female	33	No	Congenital heart disease	7	2.6	1.1	0.9	NA	Normal (90% of the control)	Array CGH (prenatal study)	No	Death at 5 months of age
Case 2017-15<br><br>22q11.2 deletion	Female	37	No	Hypoparathyroidism	13	5.1	1.7	1.5	NA	Normal (74% of the control)	Array CGH	No	Alive (8 y) without significant complications
Case 2017-16<br><br>22q11.2 microdeletion and ataxia telangiectasia	Female	41	No	No	14	1.7	1.2	0.8	NA	Normal (75% of the control)	Exome and array CGH	No	Alive (8 y) without significant complications.
Case 2018-5<br><br>*PTEN* Hamartoma Tumor Syndrome	Male	35	No	Macrocephaly	7	2.8	1.8	1.5	53	Normal (75% of the control)	Exome	Upper respiratory infections	Alive (7 y) without significant complications.

Case 2018-7<br><br>Down syndrome	Female	31	No	Esophageal atresia type 1, congenital hypothyroidism	13	1.9	1.2	0.8	NA	Normal (80% of the control)	Karyotype (prenatal study)	No	Alive (7 y). Pulmonary hypertension, craniosynostosis and recurrent bronchitis.
Case 2019-4<br><br>SHORT syndrome	Male	37	No	Intrauterine growth retardation	7	5.5	1.7	1.3	89	Normal (60% of the control)	Sanger	No	Alive (7 y) syndromic phenotype, sensorineural hearing loss, neurodevelopmental delay
Case 2019-11<br><br>Unclassified syndromic patient	Female	38	No	Spina bifida, hydrocephalus and congenital hypothyroidism	16	5	1.8	1.1	83	Normal (63% of the control)	Array CGH, IEI gene panel and exome	Uncomplicated urinary tract infection	Alive (6 y) without significant complications..
Case 2019-12<br><br>22q11.2 deletion	Male	40	No	No	8	3.3	1.2	0.8	81	Normal (90% of the control)	Array CGH		Alive (6y) without significant complications..
Case 2020-3<br><br>22q11.2 deletion	Female	39	No	No	16	3.3	1.2	0.8	64	Normal (90% of the control)	Array CGH	No	Alive (6 y) without significant complications..
Case 2020-7<br><br>22q11.2 deletion	Male	38	No	Corneal opacity	9	3.3	0.7	0.5	73	Normal (100% of the control)	Array CGH	No	Alive (5 y) without significant complications..
Case 2020-10<br><br>22q11.2 deletion	Female	40	No	Cleft palate	16	5.6	1	0.7	68	Normal (100% of the control)	Array CGH	No	Alive (5 y) without significant complications..

Case 2021-3<br><br>Unclassified syndromic patient	Female	33	Yes	Brain, heart, and genital malformations	3	0.9	0.6	0.3	33	NA	Array CGH and Exome	No	Death at 2 weeks of age
Case 2021-9<br><br>Ataxia telangiectasia	Female	40	Yes	No	10	1.6	0.7	0.4	49	Normal (86% of the control)	Array CGH and IEI gene panel	No	Alive (4 y) without significant complications
Case 2021-11<br><br>Down syndrome	Female	33	No	Chylothorax and congenital heart disease	6	0.9	0.6	0.3	24	Normal (55% of the control)	Karyotype (prenatal study)	No	Death at 2 months of age
Case 2021-12<br><br>Down syndrome	Female	37	No	Duodenal and esophageal atresia	12	2	1.1	0.8	68	Normal (100% of the control)	Karyotype (prenatal study)	No	Alive (4 y) without significant complications
Case 2021-14<br><br>22q11.2 deletion	Female	38	No	No	3	2.8	0.5	0.4	63	Normal (55% of the control)	Array CGH	No	Alive (4 y) without significant complications
Case 2021-15<br><br>Unclassified syndromic patient	Male	40	Yes	Congenital cataract	8	3.4	1.5	1.1	80	Normal (70% of the control)	Array CGH, IEI gene panel and exome	No	Alive (4 y) without significant complications
Case 2021-16<br><br>22q11.2 deletion	Male	37	No	Bilateral hydronephrosis and ventricular septal defect	12	4.4	1.3	0.9	73	Normal (77% of the control)	Array CGH	No	Alive (4 y) without significant complications
Case 2022-2<br><br>22q11.2 deletion	Male	37	No	Hypoparathyroidism and intestinal volvulus	4	2	0.9	0.6	71	Normal (90% of the control)	Array CGH (prenatal study)	Upper respiratory infections	Alive (4 y) without significant complications

Case 2022-9<br><br>Down syndrome	Male	39	No	Congenital heart disease	7	2.1	1	0.77	74	Normal (85% of the control)	Karyotype	No	Alive (3 y) without significant complications
Case 2022-11<br><br>Ataxia telangiectasia	Male	38	No	No	10	2.8	0.9	0.6	62	Normal (79% of the control)	IEI gene panel	No	Alive (3 y) without significant complications
Case 2022-13<br><br>22q11.2 deletion	Male	38	No	No	14	3.2	1.4	0.9	64	Normal (85% of the control)	Array CGH	No	Alive (3 y) without significant complications
Case 2022-14<br><br>Ataxia telangiectasia	Female	40	No	No	4	1.6	0.9	0.6	45	Normal (90% of the control)	IEI gene panel	No	Alive (3 y) without significant complications
Case 2023-11<br><br>Unclassified syndromic patient	Male	39	No	Pulmonary sling and liver disease	5	4.3	1.4	1	74	Normal (90% of the control)	Array CGH and exome	Upper respiratory infections	Alive (2 y) without significant complications

CGH, Comparative genomic hybridization;*CRU*, Clinical Reference Unit; *GA*, Gestational age; *IEI*, Inborn error of immunity; *NA*, Not available; *PHA*, Phytohemagglutinin; *RI*, Reference interval; *TREC*, T-cell receptor excision circle.

Table S3. Data from first SCID-CRU visit and clinical/immunological outcome of all patients with idiopathic lymphopenia.

**Case ID**	**Sex**	**GA (weeks)**	**History of consanguinity**	**Infections or other clinical manifestations at the beginning of the study**	**TREC (copies/µL)** Cut-off ≥20	**Lymphocyte count ($\times 10^9$/L)** (RI: 3.4 – 7.6)	**T-cell lymphocyte count ($\times 10^9$/L)** (RI: 1.85 – 5.96)	**CD4+ T-cell lymphocyte count ($\times 10^9$/L)** (RI: 1.14 – 3.80)	**CD4+ naive (%)** (RI: 54–80)	**PHA lymphocyte proliferation** (RI: >50% control)	**Genetic studies**	**Infections during follow-up**	**Outcome and current status**
Case 2017-4	Male	40	No	No	14	3.7	2.2	1.6	NA	Normal (84% of the control)	Array CGH, IEI gene panel and exome (negative results)	No	Alive (8 y) with severe autism spectrum disorder. No lymphopenia since the age of 6 years.
Case 2017-11	Male	40	No	No	7	3.3	1.7	1.5	80	Normal (80% of the control)	Array CGH and IEI gene panel (negative results)	No	Alive (7 y) and asymptomatic with persistent CD3+ and CD4+ lymphopenia. No prophylaxis or contraindication to live vaccines.
Case 2018-9	Male	39	No	No	10	3.7	1.1	0.7	81	Normal (80% of the control)	Array CGH and IEI gene panel (negative results)	No	Alive (6 y) and asymptomatic with persistent CD3+ and CD4+ lymphopenia. No prophylaxis or contraindication to live vaccines.

Case 2019-3	Female	39	No	No	9	2.8	1.4	1.1	79	Normal (100% of the control)	Array CGH and IEI gene panel (negative results)	No	Alive (6 y) and asymptomatic. No lymphopenia since the age of 1 year.
Case 2019-5	Male	39	No	No	13	2.9	1.9	1.2	70	Normal (100% of the control)	IEI gene panel (*AIRE* c.G901A / p.V301M in heterozygosis)	No	Alive (6 y) and asymptomatic with persistent CD4+ lymphopenia. No prophylaxis or contraindication to live vaccines.
Case 2020-5	Female	38	No	Congenital toxoplasmosis	4	1.8	1	0.4	49	Abnormal (18% of the control)	Array CGH, IEI gene panel and exome (negative results)	Uncomplicated horizontal CMV infection	Alive (5 y) with onset of vascular malformations and persistent CD3+ lymphopenia with normal PHA. No prophylaxis or contraindication to live vaccines.
Case 2020-9	Male	40	No	No	13	6.6	1.7	1.1	72	Normal (100% of the control)	Array CGH (negative results)	No	Alive (4 y) and asymptomatic. No lymphopenia since the age of 1 year.
Case 2021-1	Female	40	No	No	14	3.1	1.3	0.7	79	Normal (100% of the control)	Not performed	No	Alive (4 y) and asymptomatic. No lymphopenia since the age of 3 years.

Case 2021-4	Male	40	No	No	5	3.6	1.5	0.9	62	Normal (80% of the control)	Array CGH and IEI gene panel (negative results)	No	Alive (4 y) and asymptomatic with persistent CD3+ and CD4+ lymphopenia. No prophylaxis or contraindication to live vaccines.
Case 2021-10	Female	37.5	No	No	0	1.1	0.47	0.3	53	Normal (90% of the control)	IEI gene panel (*AIRE* c.G901A / p.V301M in heterozygosis)	Uncomplicated urinary tract infection	Alive (3 y) and asymptomatic with persistent CD3+ and CD4+ lymphopenia. Cotrimoxazole prophylaxis and contraindication of live vaccines.
Case 2022-4	Male	38	No	No	16	2.9	1.05	0.7	70	Normal (80% of the control)	Array CGH and IEI gene panel (negative results)	No	Alive (3 y) and asymptomatic with persistent CD3+ and CD4+ lymphopenia and suspected autoimmune neutropenia. No prophylaxis or contraindication to live vaccines.
Case 2022-6	Male	41	No	No	6	1.2	0.6	0.4	76	Normal (92% of the control)	Array CGH, IEI gene panel and exome (negative results)	Uncomplicated horizontal CMV infection	Alive (3 y) and asymptomatic with persistent CD3+ and CD4+ lymphopenia. No prophylaxis or contraindication to live vaccines.

Case 2022-8	Female	38	No	No	8	2.5	1.2	1	66	Normal (70% of the control)	Array CGH and IEI gene panel (negative results)	Uncomplicated viral respiratory infections	Alive (3 y) and asymptomatic with persistent CD3+ and CD4+ lymphopenia. No prophylaxis or contraindication to live vaccines.
Case 2023-1	Male	40	Si	No	16	3.7	1.6	1.2	89	Normal (97% of the control)	Array CGH and IEI gene panel (negative results)	No	Alive (2 y) and asymptomatic with persistent CD3+ and CD4+ lymphopenia. No prophylaxis or contraindication to live vaccines.
Case 2023-7	Male	38	No	No	14	2	0.9	0.5	15	Absent (10% of the control)	Array CGH and IEI gene panel (negative results)	No	Alive (1.5 y) and asymptomatic with persistent CD3+ and CD4+ lymphopenia. No prophylaxis or contraindication to live vaccines.

CGH, Comparative genomic hybridization; *CMV*, Cytomegalovirus; *CRU*, Clinical Reference Unit; *GA*, Gestational age; *IEI*, Inborn error of immunity; *NA*, Not available; *PHA*, Phytohemagglutinin; *RI*, Reference interval; *SCID*, Severe combined immunodeficiency; *TREC*, T-cell receptor excision circle.
